# High-flow nasal cannula: Evaluation of the perceptions of various performance aspects among Chinese clinical staff and establishment of a multidimensional clinical evaluation system

**DOI:** 10.3389/fmed.2022.900958

**Published:** 2022-07-15

**Authors:** Ruoxuan Wen, Xingshuo Hu, Tengchen Wei, Kaifei Wang, Zhimei Duan, Zhanqi Zhao, Lixin Xie, Fei Xie

**Affiliations:** ^1^College of Pulmonary and Critical Care Medicine, Chinese PLA General Hospital, Beijing, China; ^2^Department of Respiratory Medicine, 907 Hospital of the Joint Logistics Team, Nanping, China; ^3^Institute of Technical Medicine, Furtwangen University, Villingen-Schwenningen, Germany

**Keywords:** clinical evaluation system, high-flow nasal cannula, education, temperature accuracy, humidification capacity, flow rate accuracy, oxygen concentration accuracy

## Abstract

**Objective:**

In order to facilitate education for clinical users, performance aspects of the high-flow nasal cannula (HFNC) devices were evaluated in the present study. A multidimensional HFNC clinical evaluation system was established accordingly.

**Materials and Methods:**

Clinical staff from Chinese hospitals were invited to participate in an online questionnaire survey. The questionnaire was mainly about the accuracy of temperature, flow rate, and oxygen concentration of HFNC, as well as its humidification capacity. We also investigated how the clinical staff of different professions made decisions on HFNC evaluation indicators. Based on the results of the questionnaire survey of clinicians with rich experience in using HFNC, the relative weights of temperature accuracy, flow velocity accuracy, oxygen concentration accuracy, and humidification ability of HFNC equipment were calculated by the AHP to establish a clinical evaluation system. Four kinds of common HFNC devices were tested and evaluated, and the clinical performance of the four kinds of HFNC devices was evaluated by the new scoring system.

**Results:**

A total of 356 clinicians participated in and completed the questionnaire survey. To ensure the reliability of the HFNC evaluation system, we only adopted the questionnaire results of clinicians with rich experience in using HFNCs. Data from 247 questionnaires (80 doctors, 105 nurses, and 62 respiratory therapists [RTs]) were analyzed. A total of 174 participants used HFNC more than once a week; 88.71% of RTs used HFNC ≥ 1 score daily, 62.86% of nurses used HFNC ≥ 1 score daily, and 66.25% of doctors used HFNC ≥ 1 daily. There was no significant difference in the frequency of use between doctors and nurses. Finally, the relative weights of temperature accuracy (0.088), humidification capacity (0.206), flow velocity accuracy (0.311), and oxygen concentration accuracy (0.395) in the HFNC clinical evaluation system were obtained. The relative weights of clinicians with different occupations and the frequency of HFNC use were obtained. After testing four kinds of HFNC devices through the evaluation system, it was found that the four kinds of HFNC devices have different advantages in different clinical performances, and AiRVO_2_ has excellent performance with regard to temperature accuracy and humidification ability. HF-75A and NeoHiF-i7 are good at ensuring the stability of oxygen concentration and the accuracy of the flow velocity of the transported gas, while OH-80S is relatively stable in all aspects.

**Conclusion:**

The clinical evaluation system of HFNC is based on the weight of the experience of clinical personnel with different medical backgrounds. Although the existing practitioners have different educational backgrounds (academic qualifications, majors), our evaluation system can enhance clinical staff’s awareness of HFNC and further optimize the clinical use of HFNC.

## Introduction

High-flow nasal cannula (HFNC) oxygen therapy is a novel oxygen therapy involving the administration of inhaled oxygen at a constant temperature, flow rate, and oxygen concentration through nasal plugs. It is currently among the common oxygen therapies for critically ill patients in the intensive care unit (ICU) (1–3). HFNC has a good therapeutic effect on patients with acute respiratory failure and is effective for patients with mild acute respiratory distress syndrome (4, 5). Further clinical research has allowed the application of HFNC in numerous clinical settings, including hypercapnia-induced respiratory failure, pre-oxygenation before intubation, and post-extubation continuous supportive (6–8). Recently, HFNC has been found to reduce the rate of tracheal intubation and mortality in patients with acute respiratory failure due to coronavirus disease 2019 (COVID-19) (9).

Given the increasing popularity of HFNC in clinical practice, many companies have begun manufacturing HFNC devices. The various brands of HFNC devices have differences in humidification capacity, product stability and reliability, and water accumulation in the breathing circuits. Most studies on the performance of HFNC devices have focused on a single performance characteristic, including the humidification capacity or factors affecting the actual oxygen concentration (10–12). Accordingly, there have been no studies performing a comprehensive clinical evaluation of the overall performance of HFNC devices. Moreover, most studies have used the same brand of HFNC devices, with no comprehensive comparisons among multiple brands; therefore, current evidence on HFNC devices is one-sided (10, 11, 13, 14). This impedes clinical staff from elucidating the characteristics of the different brands of HFNC devices, and therefore choosing the appropriate HFNC devices based on the clinical needs.

Therefore, comprehensively evaluating the clinical performance of HFNC equipment and understanding the characteristics of the different brands of HFNC equipment is particularly important. Because of the multiple evaluation indexes involved in the comprehensive evaluation of the clinical performance of HFNC, we can decompose the indicators into multiple levels by using the analytic hierarchy process (AHP) and carry out qualitative and quantitative analysis, to get a more scientific weight system (15).

The purpose of this study was to understand the decisions made by Chinese clinicians of different occupations, workplaces, and frequencies of use of HFNC in choosing different brands of HFNC devices and using HFNC. Based on this, we aimed to establish a multi-angle clinical evaluation system of HFNC to evaluate the clinical performance of four commonly used brands of HFNC devices to help clinicians choose the appropriate HFNC devices and treatment strategies according to different clinical needs.

## Materials and Methods

**FIGURE 1:**
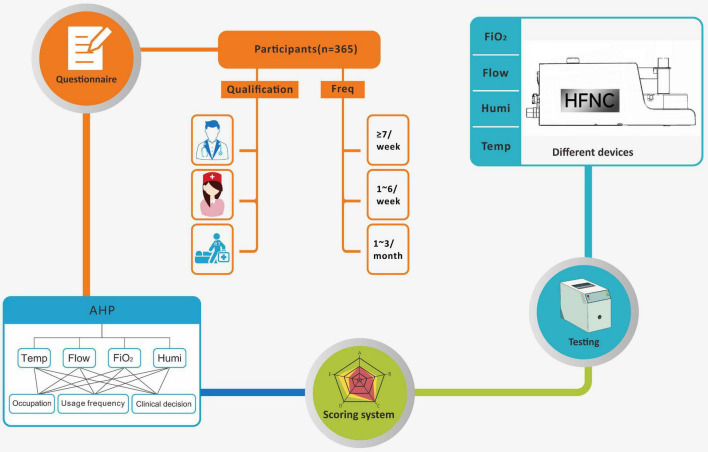
HFNC clinical evaluation system method for establishing and testing.

Figure 1 shows the experimental process of our entire study. We used AHP to establish the clinical evaluation system of HFNC (15, 16). According to our experience and previous clinical studies (17), we set temperature, velocity accuracy, oxygen concentration accuracy, and humidification ability as the basic components of the HFNC clinical evaluation system, took the positive and negative values of temperature, flow rate, and oxygen concentration accuracy as further evaluation indexes, and finally formulated clear and quantifiable evaluation criteria. A questionnaire was designed for the identified indicators, and an online questionnaire survey was conducted among Chinese clinical staff (Supplementary Material 1). Finally, the questionnaire survey results of the clinicians with a rich experience in using HFNC were selected, the comparison of the importance between the two indicators at the same level was determined, and the judgment matrix was obtained. Using the judgment matrix, the preference of clinicians in the face of the deviation between the actual measured value and the preset value was further compared. If the clinician was more able to accept that the deviation value was positive, the score increased when the deviation value was positive. When the deviation value was negative, the scores of all indicators were normalized to get the weight of each index.

Subsequently, the established HFNC clinical evaluation system was used to assess four common brands of HFNC devices in Chinese hospitals, namely, AiRVO_2_ (Fisher & Paykel Healthcare, Auckland, New Zealand), HF-75A (YUWELL, Guangzhou, China), NeoHiF-i7 (Aeonmed, Hebei, China), and OH-80S (Microme, Hunan, China). Supplementary Material 2 shows the specific performance parameters of the four HFNC devices.

The performance assessment was conducted in a laboratory (Beijing, China) with an ambient temperature of 26°C and relative humidity of 30%. During the test, a stabilization time of 15 min was allowed after each change of the HFNC parameter settings. Subsequently, data were recorded at 10-s intervals within 1 min, with the average values being used for analysis. Additionally, we examined the amount of condensed water in the breathing circuits of the four HFNC devices (Supplementary Material 3B). The details of the test setup are described as follows:

### Temperature accuracy

For each device, the temperature parameter was set at 31, 34, and 37°C; moreover, the actual temperature was individually measured. At each temperature setting, three different flow rates (20, 40, and 60 L/min) were set in that order, with the HFNC device being stabilized for 15 min after each parameter change. The actual temperature of the delivered gas was measured using a contact thermometer (YHT309, Yuanhengtong Technology, Shenzhen, China) at the nasal cannula(➀, Supplementary Material 3A). The temperature accuracy was evaluated by the relative temperature accuracy; The measured value of relative temperature accuracy in the range of 0–100. The temperature accuracy full score was 100, the higher the temperature relative accuracy score was, the higher the final score was. This was followed by the evaluation of the relative temperature accuracy as follows: (1 – | (T_measured_ – T_set_)| /T_set_)*100%, where T_measured_ and T_set_ were the measured and set temperatures, respectively.

### Humidification capacity

Using a hygrometer (VICTOR 231, Double King Industrial Holdings, Shenzhen, China), we measured the relative humidity of the delivered gas at 31, 34, and 37°C at the nasal cannula of each HFNC device (➀, Supplementary Material 3A), followed by matching of three different flow rates as previously described, with stabilization for 15 min after each parameter change. Humidification capability was directly evaluated as the relative humidity of the delivered gas. The humidification ability was directly evaluated by the relative humidity of the output gas, and the measured value of relative humidity fluctuated in the range of 0–100. The full score of the humidification ability was 100; The higher the relative humidity of the delivered gas was, the higher the final score was.

### Flow rate accuracy

Each HFNC device was set to three flow rates (20, 40, and 60 L/min), followed by incremental matching with three oxygen concentrations (30 60, and 90%). The HFNC device was stabilized for 15 min after each parameter change. The actual flow rate of the delivered gas was measured using a gas flow meter (MF5612-N-200-AB-D-A, Xiargo Micro Electromechanical System, Sichuan, China) at the circuit outlet (➁, Supplementary Material 3A). The flow rate accuracy was evaluated by the relative flow rate accuracy; The measured value of relative flow rate accuracy in the range of 0–100. The flow rate accuracy full score was 100, the higher the flow rate relative accuracy score was, the higher the final score was. This was followed by the evaluation of the relative flow rate accuracy as follows: (1 – | (Flow_measured_ – Flow_set_)| /Flow_set_)*100%, where Flow_measured_ and Flow_set_ were the measured and set flow rates, respectively.

### Oxygen concentration accuracy

Each HFNC device was set to three oxygen concentrations (FiO_2_; 30, 60, and 90%), which were each incrementally matched with three flow rates (20, 40, and 60 L/min). The HFNC device was stabilized for 15 min after each parameter change. The actual oxygen concentration of the delivered gas was measured using a stationary medical oxygen analyzer (MOT500-O2-M-CL, Korno electronic technology, Shenzhen, China) at the breathing circuit outlet (➁, Supplementary Material 3A). The oxygen concentration accuracy was evaluated by the relative oxygen concentration accuracy; The measured value of relative oxygen concentration accuracy in the range of 0–100. The oxygen concentration accuracy full score was 100, the higher the oxygen concentration relative accuracy score was, the higher the final score was. Finally, the relative accuracy of oxygen concentration was calculated as follows: (1 – | (FiO_2measured_ – FiO_2set_)| /FiO_2set_)*100%, where FiO_2measured_ and FiO_2set_ were the measured and set oxygen concentrations, respectively.

### The amount of accumulated condensed water

Each HFNC device was equipped with 2 L of sterile humidification water. Moreover, the temperature, flow rate, and FiO_2_ were uniformly set to 37°C, 20 L/min, and 21%, respectively. The breathing circuit and nasal cannula of the HFNC device were weighed before the operation (Vol_1_, Supplementary Material 3B) and after 6 h of continuous device operation (Vol_2_, Supplementary Material 3B). Finally, the amount of accumulated condensed water was calculated as follows: Vol_2_-Vol_1_.

### Statistical analysis

The data are presented as mean ± SD. The chi-Square test was used for categorical variables where applicable. A *p* value < 0.05 was considered statistically significant.

## Results

Clinical staff from hospitals in Sichuan, Tibet, Fujian, Beijing, Zhejiang, etc., 11 places in China in total, were invited to participate in an online questionnaire survey, with 1,015 questionnaires distributed. The questionnaires were completed by 356 clinical staff members. To ensure the reliability of the HFNC evaluation system, we only adopted the results of the questionnaires from clinicians who had a rich experience in using HFNC (using HFNC ≥ 1/week). We excluded 109 questionnaires and eventually included 247 questionnaires in the study.

**FIGURE 2:**
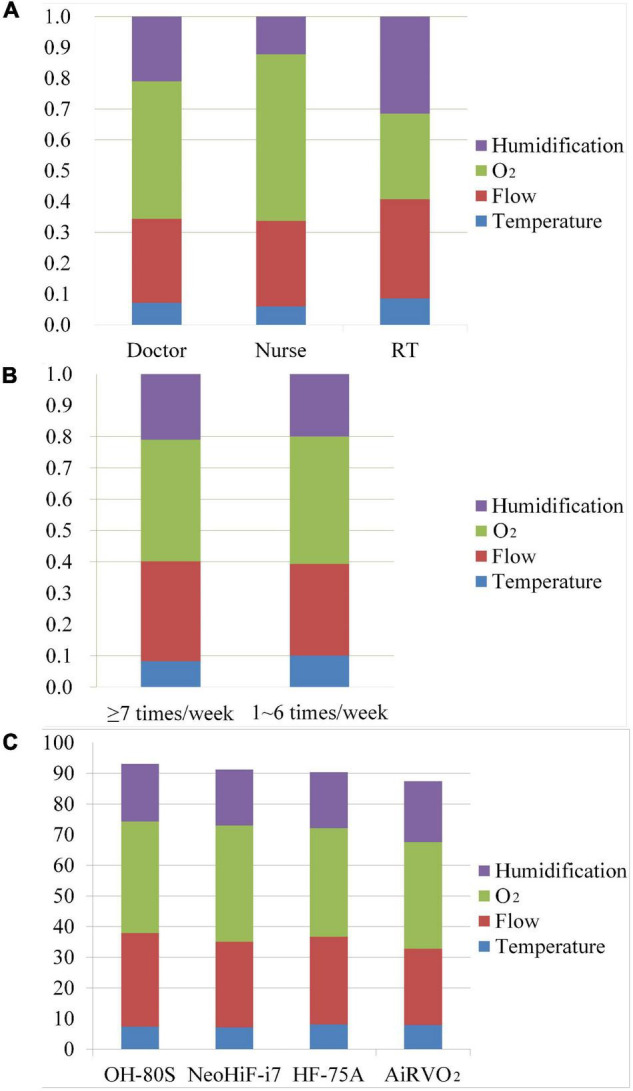
The comparison of different occupation usage frequency and HFNC devices in the HFNC clinical evaluation system. **(A)** The comparison of the weights of doctors, nurses, and RTs in the HFNC clinical evaluation system. **(B)** The weights of different usage frequencies of HFNC in the HFNC clinical evaluation system. Each histogram consists of weight coefficients of four aspects in the HFNC clinical evaluation system. **(C)** The clinical performance of four HFNC devices. Humidification: humidification capacity. O_2_: oxygen concentration accuracy. Flow: flow rate accuracy. Temperature: temperature accuracy.

Table 1 and Figure 2A shows the relationship between different occupations and the frequency of using HFNC. The 247 participants included 80 doctors, 105 nurses, and 62 respiratory therapists (RT). Of the clinicians enrolled in this study, 70.4% used HFNC more than once a day. The study found that 88.71% of RT used HFNC daily, which was significantly different from the frequency of doctors and nurses using HFNC (*P* < 0.001). There was no statistical difference in the proportion of doctors and nurses using HFNC per day (66.25 vs. 62.86%).

**TABLE 1:** Relationship between occupation and usage frequency of high-flow nasal cannula (HFNC).

Occupation	Number (n)	Usage frequency of HFNC	
		≥ 7 times/week	1∼6 times/week
Doctor	80	66.25%	33.75%
Nurse	105	62.86%	37.14%
Respiratory therapist (RT)	62	88.71%	11%

Further analysis showed that the relative weights of the four parts of the HFNC clinical evaluation system were affected by occupation (Table 2). The three occupations had different emphases on each evaluation part of HFNC4. Obviously different from RT, doctors and nurses thought that the accuracy of oxygen concentration was the most important aspect, with a relative weight of 0.541 and 0.447, respectively (*P* < 0.001), but paid different attention to the accuracy of flow velocity and humidification ability. Compared with nurses, doctors thought that the accuracy of oxygen concentration was the most important aspect, but the emphasis on the four aspects of evaluation was more balanced. For example, doctors also valued the accuracy of flow velocity and humidification ability (0.272 vs. 0.209) of HFNC, while nurses attached much less importance to the accuracy of flow rate and humidification ability than oxygen concentration accuracy (0.277 vs. 0.123 vs. 0.541). RTs had the most balanced emphasis on oxygen concentration accuracy, flow velocity accuracy, and humidification capacity, with relative weights of 0.277, 0.323, and 0.315, respectively. However, relatively speaking, they paid more attention to the accuracy of flow velocity and humidification ability.

**TABLE 2:** Influence and relative weights of different occupations and usage frequency of high-flow nasal cannula (HFNC) on the importance of all aspects of the HFNC clinical evaluation system.

	Doctor	Nurse	RT	≥ 7 times/week	1∼6times/week	P (occupation)	P (usage frequency)
Number (n)	80	105	62	174	73		
Temp vs. F	22	18	14	35	19	p = 0.237	p = 0.305
	58	87	48	139	54		
Temp. vs. O_2_	19	11	17	33	14	p = 0.342	p = 0.969
	61	18	45	141	59		
F vs. O_2_	39	26	35	74	26	p < 0.001	p = 0.313
	41	79	27	100	47		
Hum. vs. Temp.	53	69	52	127	47	p = 0.028	p = 0.176
	27	36	10	47	26		
Hum vs. F	36	25	30	66	25	p = 0.001	p = 0.584
	44	80	32	108	48		
Hum vs. O_2_	37	25	28	61	29	p = 0.002	p = 0.487
	43	80	34	113	44		

	**Relative weights**					**Weight**	

Temp	0.072	0.060	0.086	0.083	0.101	0.088	
Hum	0.209	0.123	0.315	0.209	0.199	0.206	
F	0.272	0.277	0.323	0.319	0.293	0.311	
O_2_	0.447	0.541	0.277	0.389	0.408	0.395	
Total weight	1	1	1	1	1	1	

*Temp: temperature accuracy; Hum: humidification capacity; F: flow rate accuracy; O2: oxygen concentration accuracy.*

The relative importance of the four performance aspects of the HFNC devices differed according to profession (Table 2). Nurses considered the accuracy of oxygen concentration as the most important performance aspect; moreover, their degree of emphasis on each performance aspect significantly differed from those of doctors and RTs (*P* < 0.001). Additionally, nurses placed significantly more emphasis on the flow rate accuracy than the humidification capacity (*P* < 0.001). Doctors and RTs also showed biases in the four performance aspects. For example, doctors placed more regard on oxygen concentration accuracy and flow rate accuracy, while RTs placed more regard on the flow rate accuracy and humidification capacity. However, doctors and RTs showed a more balanced opinion regarding oxygen concentration accuracy, flow rate accuracy, and humidification capability.

**FIGURE 3:**
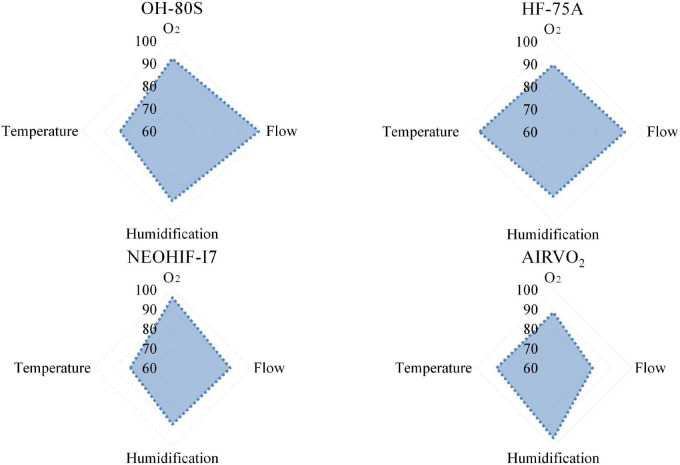
A radar chart of four HFNC devices’ clinical performances. According to the scores calculated by the HFNC clinical evaluation system, the total score of each item is 100, and the total score is 400.

There was no correlation between the usage frequency of HFNC and the relative weights for each performance aspect (Table 2). Moreover, the perceptions of the different performance aspects did not significantly differ according to the proficiency levels in using HFNC (Figure 3B).

Finally, the relative weights of the HFNC clinical evaluation system were determined based on survey results, as well as how they differed according to profession and frequency of HFNC usage (Table 2), which was displayed in a histogram (Figure 3).

### Humidification capability and temperature accuracy

Compared with other performance aspects, humidification capacity and temperature accuracy received less attention, with weights of 0.204 and 0.091, respectively. Compared with the deviation of the actual flow rate from the preset value, the actual output temperature being lower than the preset value was considered more acceptable by the clinical staff. Furthermore, 20.2 and 79.8% of the clinical staff accepted the actual temperature being higher and lower, respectively, than the preset value.

### Oxygen concentration accuracy

Most clinical staff considered oxygen concentration accuracy as the most important performance aspect, with a weight of 0.405. In case of deviations, 66.9 and 33.1% of the clinical staff preferred that the actual oxygen concentration was higher and lower, respectively, than the preset value.

### Flow rate accuracy

The relative weight for the flow rate accuracy was 0.299. In case of deviations, 64 and 36% of the clinical staff preferred that the actual flow rate was higher and lower, respectively, than the preset value.

### High-flow nasal cannula device evaluation

After establishing the HFNC clinical evaluation system, four common brands of HFNC devices were evaluated. We demonstrated the performance of four HFNC devices from two angles. We tested the temperature accuracy, humidification capacity, flow rate accuracy, and oxygen concentration accuracy of the four HFNC devices. The full score of each aspect was 100. Using the radar chart in Figure 3, we directly show the characteristics of the four HFNC devices. Among them, the advantage of AiRVO_2_ lay in the temperature accuracy and humidification ability when conveying gas. HF-75A and NeoHiF-i7 were good at ensuring the stability and accuracy of oxygen concentration in gas transport, while OH80S was stable in all aspects. We then used the HFNC clinical evaluation system to calculate the test data and the final score of each device according to the weight, as well as show the overall performance of the four kinds of HFNC devices in the form of a bar chart (Figure 2C). Among them, NeoHiF-i7 was the best in the accuracy of oxygen concentration and OH-80S was outstanding in the accuracy of velocity. AiRVO_2_ had the best performance in temperature accuracy and humidification ability.

The amounts of accumulated condensed water in AiRVO_2_, OH-80S, and HF-75A were 16, 21, and 1 g, respectively. No condensed water was found in NeoHiF-i7.

## Discussion

This is the first questionnaire survey on the opinions and biases of various clinical staff with respect to four performance aspects of HFNC devices. An HFNC clinical evaluation system was subsequently established and used to evaluate four brands of HFNC devices. There were no significant differences in the perception of all performance aspects according to the usage frequency of HFNC devices. However, there were differences according to profession, with nurses showing significantly different perspectives from those of doctors and RTs.

### Perceptions of high-flow nasal cannula performance aspects by different clinical staff

Nurses considered oxygen concentration accuracy as the most important performance aspect, followed by flow rate accuracy. This could be attributed to the daily work of nurses. First, nurses mainly work at the bedside and have the closest contact with patients; therefore, they are often the first to detect deterioration in the patient’s condition. In case of sudden deterioration, increasing the oxygen concentration is among the effective relief measures. Moreover, since the actual oxygen concentration delivered to the patient is dependent on the patient’s inspiratory flow rate and the flow rate of the delivered gas (18), the flow rate accuracy indirectly affects the inhaled oxygen concentration. Additionally, the flow rate of the delivered gas directly affects the patient’s comfort during the use of HFNC. If the flow rate is set too high, the gas flow rate will be too fast. Contrastingly, if the flow rate is set too low, the patient will feel suffocated. Both situations aggravate the patient’s intolerance to HFNC, which results in mouth breathing and even refusal to use the HFNC device. Several nurses take turns in taking care of one patient; therefore, nurses pay more attention to short-term, rather than long-term, changes in the patient’s condition. Changes in oxygen concentration have short-term and rapid effects on the patient’s condition. Taken together, these factors explain why compared with doctors and RTs, nurses paid more attention to oxygen concentration accuracy and flow rate accuracy.

Contrastingly, doctors and RTs are usually individually responsible for the entire treatment process, from admission to recovery and discharge. Accordingly, they are more likely to evaluate patients on HFNC therapy from a comprehensive and long-term perspective. Based on their role, doctors pay more attention to the clinical indications and contraindications of HFNC therapy (17), parameter adjustment during HFNC use for patients with different diseases, and the impact of HFNC therapy on clinical outcomes and mortality (19). For example, doctors consider the timing of starting HFNC therapy in patients with type I respiratory failure; how to predict HFNC failure through the patient’s consciousness, arterial blood gas analysis, and other indicators (17, 20); and how to adjust specific HFNC parameters for patients with type II respiratory failure (21). Therefore, doctors were most concerned about the accuracy of oxygen concentration of HFNC to ensure the safety and effectiveness of HFNC. At the same time, because of the need to comprehensively consider HFNC use from multiple perspectives. Compared with nurses, doctors showed relatively balanced attention to the flow rate accuracy, oxygen concentration accuracy, and humidification capacity, which influence the treatment outcome (Figure 2B).

Respiratory therapists (RTs) were most concerned about the treatment and adverse effects of HFNC use. Compared with doctors and nurses, RTs paid more attention to the humidification capacity. Humidification capacity does not affect the patient’s short-term condition; rather, it indirectly affects the patient’s sputum characteristics, expectoration ability, and ciliary function, which influence the patient’s long-term condition (Figure 2B) (22). Since the therapeutic effect of HFNC is influenced by the patient’s comfort and cooperation, RTs evaluate the factors affecting the patient’s HFNC tolerance from multiple perspectives. Additionally, they carefully consider whether the flow rate can reach the expected value and meet the inspiratory requirements of patients with respiratory distress, whether the HFNC has excellent oxygen concentration accuracy, and whether it can provide a stable high oxygen concentration to relieve hypoxia. Therefore, similar to doctors, RTs showed relatively balanced emphases on all four performance aspects of HFNC devices.

### Implications of the findings on clinical education

Most hospitals mainly provide HFNC in ICU, while the clinical utilization rate of HFNC in non-ICU departments is low. The reason that affects the low clinical popularization rate of HFNC in non-ICU departments is that clinicians know little about HFNC technology and cannot use HFNC independently and safely in the clinic (14). Conventional HFNC training uses academic conferences, typical case discussions, teaching rounds, and other ways to help clinicians independently and safely master HFNC technology and standardize the use of HFNC, improve the success rate of HFNC use, and promote the clinical popularization rate of HFNC. However, this training method does not formulate a teaching plan according to the professional characteristics and clinical needs of clinical personnel and lacks targeted training. Clinicians need to go through a period of repeated learning and training to set individual parameters according to the needs of patients (14, 15). Although a small number of teaching methods using HFNC operation flow can help clinical staff quickly master the basic skills of using HFNC, it is difficult for beginners to use HFNC technology in complex and diverse clinical situations, flexibly set HFNC parameters, and thoroughly master HFNC technology, so the help for clinical staff is limited. Our findings could inform teaching hospitals about the initial understanding regarding HFNC technology use by the clinical staff and their focuses during its application. Accordingly, this may allow individualized education and training courses based on professional and clinical needs, which will improve the overall clinical teaching level of HFNC technology.

For bedside nurses, HFNC training courses should place more emphasis on the monitoring of HFNC therapy and operation procedures. Specifically, the nurses should be trained on the standardized protocols for the installation, use, and disinfection of HFNC devices; the relationship between the condensed water in the HFNC devices and the external temperature; and improving patient comfort with HFNC therapy (23, 24). Moreover, they should be trained on standardized HFNC wearing and breathing methods (25, 26) in order to reduce the impact of patient intolerance and mouth breathing on the actual oxygen concentration delivered by HFNC (19). In addition, they should be trained on how to analyze the factors affecting the inhaled oxygen concentration as well as the influence of the preset flow rate and oxygen concentration on the actual oxygen concentration delivered (13). Finally, they should be made aware of the actual output temperature, delivery flow rate, oxygen concentration, and amount of condensed water produced by different HFNC devices as well as the correct evaluation of the oxygenation index (PaO_2_/FiO_2_) and clinical indicators, which could improve the safety of HFNC use.

Regarding HFNC teaching for RTs, it should focus on the relevant factors that affect the therapeutic effect of HFNC and the corresponding solutions. For example, they should be taught how the gas flow rate affects the actual accuracy of oxygen concentration and relative humidity as well as the effect of the preset temperature level on the relative humidity of the delivered gas. Moreover, RTs should be trained on how to operate various common brands of HFNC devices and evaluate them using the HFNC clinical evaluation system in order to choose the appropriate HFNC device based on the specific patient needs. For example, HFNC devices with excellent humidification capacity are suitable for patients with viscous sputum that is difficult to expectorate. Our findings can be referred to when setting HFNC parameters to ensure the accuracy of the flow rate, oxygen concentration, and other indicators of the gas delivered by the HFNC device.

Training for doctors should focus on the theoretical knowledge and clinical application of HFNC technology. Initially, they can be taught the primary theoretical knowledge, including the physiological effects, indications, and contraindications of HFNC therapy. For example, HFNC therapy can improve hypoxemia in patients with type I respiratory failure by increasing the inhaled oxygen concentration, delivering high-flow gas, reducing upper airway resistance, and causing other physiological effects (1, 27). Additionally, HFNC therapy can reduce PaCO_2_ in patients with type II respiratory failure through continuous gas delivery with a high inhaled oxygen concentration and flow rate (28). Moreover, they should be trained on HFNC treatment strategies for patients with different diseases. For example, it is important to carefully consider the settings of flow rate and oxygen concentration when managing patients with chronic obstructive pulmonary disease according to their PaO_2_ and PaCO_2_. For patients with COVID-19, it is important to consider the effect of a high flow rate on the inhaled oxygen concentration and the increased risk of aerosol transmission (26). In addition, the risk of HFNC failure should be considered and addressed in patients with different respiratory conditions (20, 29, 30). Notably, there remain few hospitals with RTs. A study conducted in 2016 reported that only 32.3% of 335 Asian ICUs employed RTs (31); moreover, the duties of RTs are performed by doctors in many non-ICU departments. Therefore, training doctors regarding the primary theoretical knowledge and clinical application of HFNC technology is essential for further promoting its popularization in clinical practice.

There is a need for a closed-loop HFNC training system based on occupational characteristics, where doctors determine the HFNC treatment strategy, RTs meticulously manage HFNC use and parameter adjustment, and nurses perform standardized operations of HFNC. This could facilitate the establishment of a patient-centered HFNC treatment team.

### Evaluation of the clinical performance of high-flow nasal cannula devices

Based on our survey results, we performed a comprehensive evaluation of multiple performance aspects of HFNC devices based on the perspectives of clinical staff. Accordingly, our HFNC clinical evaluation system could help determine the optimal HFNC devices according to patient needs. With the increased clinical application of HFNC technology, several new clinical needs are being gradually explored, including improving ventilation and airway clearance in patients with cystic fibrosis as well as using it in combination with aerosolized drugs (31–33). Multidimensional assessment using our evaluation system could facilitate the improvement and development of a new generation of HFNC devices.

Evaluation of the clinical performance of different models of HFNC devices revealed model-based strengths and weaknesses in clinical performance. Therefore, our findings can help clinical staff understand the advantages of different models of HFNC devices, their suitability for patients with different conditions, and the aspects to consider in their use. Among the four models of HFNC devices, none showed the best performance in all aspects. NeoHiF-i7 showed excellent performance in oxygen concentration accuracy, which could be attributed to its use of the high-precision ultrasonic oxygen concentration flow sensor. The real-time oxygen concentration feedback and oxygen concentration control algorithm allow control of the oxygen flow required by the high-precision proportional valve output, which yields stable control of the output oxygen concentration. Moreover, the excellent performance of OH-80S in flow rate accuracy could be attributed to the use of high-precision flow sensors to detect flow. Additionally, closed-loop flow control and turbine control algorithms allow accurate and stable control of the turbine output.

AiRVO_2_ showed excellent performance in temperature accuracy and humidification capacity, which might be related to the use of high-precision temperature sensors. This detects the temperature at the end of the heating pipeline and uses real-time feedback to control the heat output of the heating wire in order to achieve precise and stable temperature control.

Notably, although different HFNC devices may use the same oxygen concentration sensor, flow sensor, and temperature sensor, they show differences in performance. This could be attributed to differences in the models and prices of high-precision sensors, which influence the final performance. Furthermore, there are among-device differences in electric circuits, software control, algorithms, etc., which influence the various performance aspects and are difficult to specify.

### Establishment of the assessment content for the high-flow nasal cannula clinical evaluation system

In our present study, we focused on 4 performance aspects. The HFNC device can continuously provide patients with warmed and humidified gas at an accurate high flow rate (0–70 L/min) and high oxygen concentration (2l–100%) (17) in order to meet the flow rate requirements during inspiration as well as reduce inspiratory resistance and respiratory work (34). Ensuring accuracy of the actual oxygen concentration of the delivered gas improves hypoxia and removes residual carbon dioxide from the physiological dead space in the nasopharyngeal cavity at the end of expiration (18, 33). In addition, humidification of the delivered gas at a preset temperature (35) reduces the impact of medical gas on the mucociliary system (36) and helps dilute sputum and maintain ciliary system function (37). Therefore, temperature stability, flow rate stability, oxygen concentration stability, and humidification capacity were considered in the evaluation system.

Besides, we evaluated the condensed water in the tube but did not include it in the evaluation system for three reasons. First, the amount of condensed water is more dependent on the breathing circuit than the device’s performance. Second, some HFNC devices can match with different brands of breathing circuits. Therefore, the amount of condensed water cannot reflect the clinical performance of the HFNC devices. Third, it remains unclear whether the amount of condensed water affects the patient’s condition. Nevertheless, the formation of condensed water in the breathing circuit and at the front end of the nasal plug is sprayed into the nostrils and causes coughing and patient discomfort (3, 24). Therefore, we performed a supplementary assessment of the amount of condensed water in the HFNC devices. There was no correlation of the amount of accumulated condensed water with the temperature accuracy and humidification capacity. The lack of a correlation between the humidification capability and amount of condensed water could be attributed to differences in the brands of breathing circuits. The material of the breathing circuit, heating wire, and pipe structure design affect the amount of condensed water (24), which could explain the aforementioned findings.

Although HFNC devices can provide a certain intraoral PEEP, factors such as sex, respiratory status, respiratory system compliance, and other factors can affect the intraoral pressure; therefore, this parameter cannot accurately reflect the performance of HFNC devices (38, 39) and was not included into the evaluation system.

### Study limitations

Our HFNC clinical evaluation system had several limitations. Our score design and evaluation content are from the point of view of clinical use and performance and are more dependent on the experience of clinical staff, but there may be some deviation. At the same time, a rich clinical experience and a solid experience in the use of HFNC can improve the success rate of HFNC treatment, so we finally included the results of the questionnaire among Chinese clinical staff with a rich experience in using HFNC (frequency ≥ 1/week). Therefore, compared with the number of people who participated in the questionnaire survey, the number of questionnaires included in the study was less. Further analysis shows that the HFNC evaluation system is affected by different occupations, so there may be biases in the survey results due to nationality and occupation distribution ratio. Therefore, we believe that the HFNC clinical evaluation system is mainly aimed at the common high-flow humidification therapeutic instruments in China, and with the further expansion of the range of people participating in the survey in the future, this problem will be solved.

## Conclusion

The clinical evaluation system of HFNC is based on the weight of HFNC experience of clinicians with different medical backgrounds to understand clinicians’ cognition of HFNC from a new perspective. Although the existing practitioners have different educational backgrounds (academic qualifications, majors), our evaluation system can enhance the clinical staff’s awareness of HFNC and help clinical staff understand the characteristics of the different brands of HFNC equipment. This may help in the training of the use of HFNC through conducting targeted teaching for participants and further optimize the clinical use of HFNC.

## Data Availability Statement

The raw data supporting the conclusions of this article will be made available by the authors, without undue reservation.

## Ethics statement

Ethical review and approval was not required for the study on human participants in accordance with the local legislation and institutional requirements. Written informed consent for participation was not required for this study in accordance with the national legislation and the institutional requirements.

## Author contributions

All authors participated in the conception and design of the manuscript and research interpretation. RW, XH, TW, KW, and ZD took the lead in data collection. LX, FX, and ZZ finish the data analysis and revised the important academic content of the manuscript. RW, XH, TW, KW, and ZD completed the first draft of the manuscript. All authors read as well as recognized the final manuscript.

## Conflict of Interest

The authors declare that the research was conducted in the absence of any commercial or financial relationships that could be construed as a potential conflict of interest.

## Publisher’s Note

All claims expressed in this article are solely those of the authors and do not necessarily represent those of their affiliated organizations, or those of the publisher, the editors and the reviewers. Any product that may be evaluated in this article, or claim that may be made by its manufacturer, is not guaranteed or endorsed by the publisher.
