# Supplementary material for: High-flow nasal cannula: Evaluation of the perceptions of various performance aspects among Chinese clinical staff and establishment of a multidimensional clinical evaluation system
Source: Front Med (Lausanne). 2022 Jul 15;9:900958. doi: 10.3389/fmed.2022.900958 (PMC9335197; doi:10.3389/fmed.2022.900958)
Supplement: Supplementary file 1 [file Data_Sheet_1.docx]

Investigation on the usage of high-flow nasal cannula

1. Profession:
   1. Doctor
   2. Nurse
   3. Respiratory therapist
   4. Other
2. Workplace：
   1. Respiratory medicine
   2. Intensive care unit
   3. Emergency department
   4. Other
3. Work seniority：
   1. Less than 1 year
   2. 1-3 years
   3. 3-5 years
   4. More than 5 years
4. The usage frequency of high-flow nasal cannula (HFNC)：
   1. Use HFNC more than 7 times/week
   2. Use HFNC 1-6 times/week
   3. Use HFNC 1-3 times/month
   4. Heard of HFNC, but never used it.
   5. Never heard of it or used it.
5. In the setting parameters of the HFNC, Temperature VS Flow rate:
   1. The former is more important
   2. The latter is more important
6. In the setting parameters of the HFNC, Temperature VS Oxygen concentration:
   1. The former is more important
   2. The latter is more important
7. In the setting parameters of the HFNC, Flow rate VS Oxygen concentration:
   1. The former is more important
   2. The latter is more important
8. For the selection of different HFNC devices, Humidification capacity VS Temperature accuracy:
   1. The former is more important
   2. The latter is more important
9. For the selection of different HFNC devices, Humidification capacity VS Flow rate accuracy:
   1. The former is more important
   2. The latter is more important
10. For the selection of different HFNC devices, Humidification capacity VS Oxygen concentration accuracy:
    1. The former is more important
    2. The latter is more important
11. When the actual temperature is different from the setting temperature:
    1. actual temperature is higher than the setting temperature.
    2. actual temperature is lower than the setting temperature.
12. When the actual flow rate is different from the setting flow rate:
    1. actual flow rate is higher than the setting flow rate.
    2. actual flow rate is lower than the setting flow rate.
13. When the actual oxygen concentration is different from the setting oxygen concentration:
    1. actual oxygen concentration is higher than the setting oxygen concentration.
    2. actual oxygen concentration is lower than the setting oxygen concentration.
