# Supplementary material for: High-flow nasal cannula: Evaluation of the perceptions of various performance aspects among Chinese clinical staff and establishment of a multidimensional clinical evaluation system
Source: Front Med (Lausanne). 2022 Jul 15;9:900958. doi: 10.3389/fmed.2022.900958 (PMC9335197; doi:10.3389/fmed.2022.900958)
Supplement: Supplementary file 2 [file Data_Sheet_2.docx]

Supplemental 2 Details of HFNC devices

|  | AiRVO_2_ | HF-75A | NeoHiF-i7 | OH-80S |
| --- | --- | --- | --- | --- |
| Temperature  Setting  (range, minimum increment) | 31,34,37°C | 31-37°C，1°C | 29-37°C，1°C | 29-37°C，1°C |
| Flow setting  (range, minimum increment) | 10–25 L/min,1 L/min;25–60 L/min,5L/min | 2–25 L/min,1 L/min;25–75 L/min,5L/min | 2–25 L/min,1 L/min;25–80 L/min,5L/min | 2–25 L/min,1 L/min;25–80 L/min,5L/min |
| Oxygen control | Oxygen flowmeter | Software setting | Software setting | Software setting |
| Oxygen setting  (range, minimum increment) | 21-95%，1% | 21-100%，1% | 21-100%，1% | 21-100%，1% |
| Heated humidifier | Built-in | Built-in | Built-in | Built-in |
| Breathing circuit | 900PT561 tube | H-180M tube | HT-08 tube | H-180M tube |
| Nasal cannula | F&P optiflow+ OPT944(medium) | NOC-06-M（medium） | Veoflo High-Flow Nasal Cannula 032-10-160CH（medium） | NOC-06-M（medium） |
