# Supplementary material for: High-flow nasal cannula: Evaluation of the perceptions of various performance aspects among Chinese clinical staff and establishment of a multidimensional clinical evaluation system
Source: Front Med (Lausanne). 2022 Jul 15;9:900958. doi: 10.3389/fmed.2022.900958 (PMC9335197; doi:10.3389/fmed.2022.900958)
Supplement: Supplementary file 3 [file Data_Sheet_3.docx]

Supplemental 3


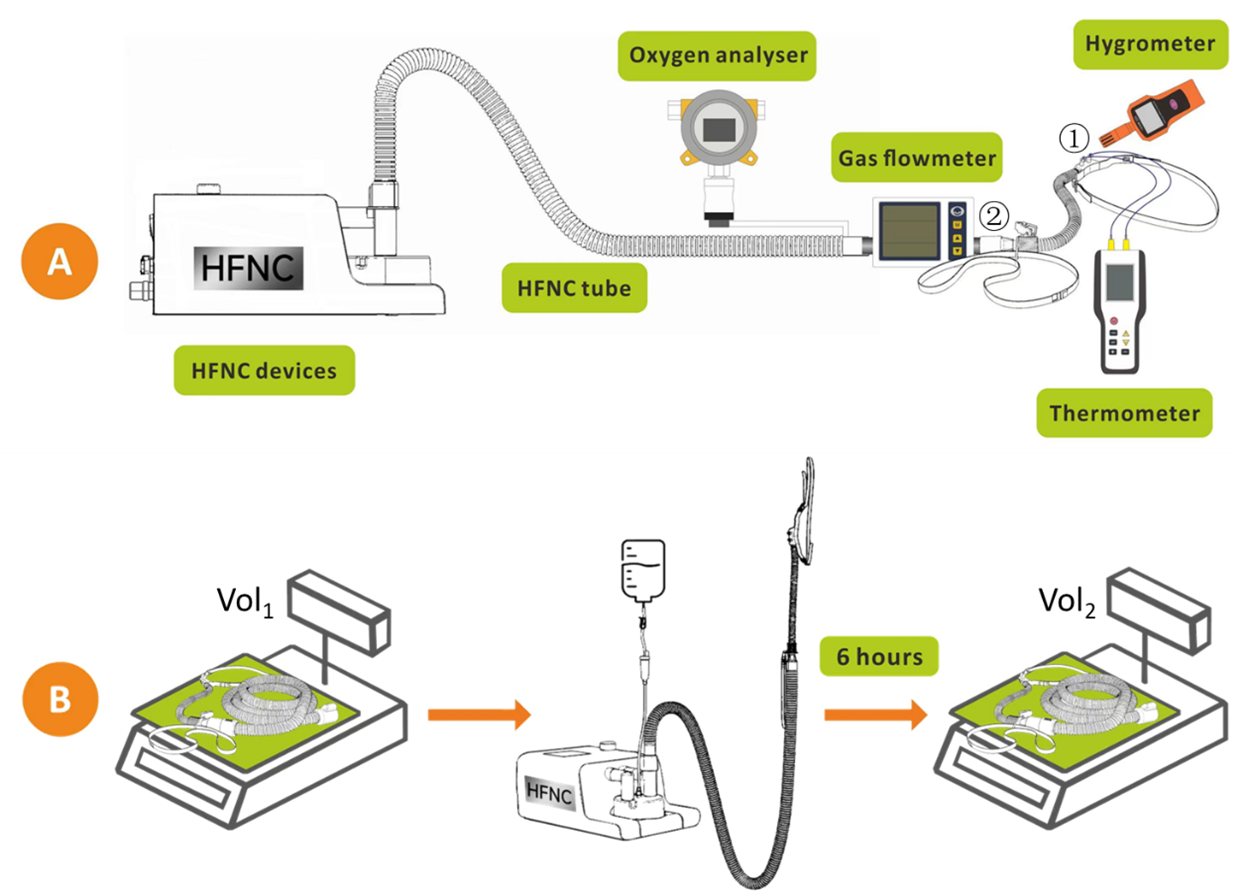


Supplemental 3：the four HFNC devices' connection and methods for testing. A: The method for testing the temperature accuracy, relative humidity, flow rate accuracy and oxygen concentration accuracy of the HFNC device. ①：the high-flow nasal cannula. ②：the breathing circuit outlet. B: The test method of condensate accumulation of four HFNC devices
